# Complaints about excessive use of police force in women’s neighborhoods and subsequent perinatal and cardiovascular health

**DOI:** 10.1126/sciadv.abl5417

**Published:** 2022-01-19

**Authors:** Alexa A. Freedman, Andrew V. Papachristos, Britney P. Smart, Lauren S. Keenan-Devlin, Sadiya S. Khan, Ann Borders, Kiarri N. Kershaw, Gregory E. Miller

**Affiliations:** 1 Department of Obstetrics and Gynecology, NorthShore University HealthSystem, Evanston, IL, USA.; 2 Institute for Policy Research, Northwestern University, Evanston, IL, USA.; 3 Department of Sociology, Northwestern University, Evanston, IL, USA.; 4 Pritzker School of Medicine, University of Chicago, Chicago, IL, USA.; 5 Department of Preventive Medicine, Northwestern University Feinberg School of Medicine, Chicago, IL, USA.; 6 Division of Cardiology, Department of Medicine, Northwestern University Feinberg School of Medicine, Chicago, IL, USA.; 7 Department of Medical Social Sciences, Northwestern University Feinberg School of Medicine, Chicago, IL, USA.; 8 Division of Maternal-Fetal Medicine, Department of Obstetrics and Gynecology, Northwestern University Feinberg School of Medicine, Chicago, IL, USA.; 9 Department of Psychology, Northwestern University, Evanston, IL, USA.

## Abstract

There are substantial, unexplained racial disparities in women’s health. Some of the most pronounced involve elevated rates of preterm delivery (PTD) and cardiovascular disease (CVD) among Black women. We hypothesized that stress associated with excessive use of force by police may contribute to these disparities. In two prospective cohorts derived from electronic health records (pregnancy cohort, *N* = 67,976; CVD cohort, *N* = 6773), we linked formal complaints of excessive police force in patients’ neighborhoods with health outcomes. Exposed Black women were 1.19 times as likely to experience PTD [95% confidence interval (CI): 1.04 to 1.35] and 1.42 times as likely to develop CVD (95% CI: 1.12 to 1.79), even after adjustment for neighborhood disadvantage and homicide. The excess risks of PTD were also observed in maternal fixed-effects analyses comparing births to the same woman. These findings suggest police violence may be an unrecognized contributor to health inequity for Black women.

## Teaser

Neighborhood police violence may contribute to increased risk of preterm birth and cardiovascular disease among Black women.

## INTRODUCTION

There are substantial and persistent racial disparities in women’s health in the United States. Two of the most pronounced, and least understood, disparities involve preterm delivery (PTD) and cardiovascular disease (CVD). Black women are over 50% more likely to deliver preterm as compared with White women, and despite multiple efforts, interventions have generally been unable to narrow this gap (*1*). Similarly, CVD prevalence is 30% higher among Black versus White women, and the avoidable death rate is nearly twice as high (*2*, *3*). Although variations in socioeconomic status (SES) contribute to these disparities, they do not fully account for them. Racial gaps are present at all levels of SES, and disparities in PTD and CVD may widen as women’s income and education increase (*4*, *5*). On top of their impact on well-being, these disparities have substantial economic implications for society, affecting labor market participation and health care expenditures and, with PTD, adversely influencing the health of the next generation (*6*–*9*).

Many of the conventional explanations for health disparities, such as access to care and healthy foods, are most applicable to women in low-SES contexts, but they are inadequate for explaining why Black women, particularly those with relatively high levels of income and education, have elevated rates of PTD and CVD, which in some cases are even greater than low-SES White women (*10*, *11*). As a result, there is increasing emphasis on the health impact of exposures that are common for, and salient to, Black women. Neighborhoods are an important focus because Black families are more likely to live in disadvantaged neighborhoods regardless of SES (*12*). As the recent deaths of Breonna Taylor, George Floyd, and others have illustrated, violent interactions with the police may be one such neighborhood exposure that may adversely contribute to health disparities.

In Chicago, which has the second-largest municipal police department in the United States, policing practices vary by neighborhood racial composition (*13*). Chicago police are nearly 10 times more likely to use force when interacting with a Black versus White individual (*14*). Moreover, unarmed Black individuals are nearly six times more likely to be shot by Chicago police as compared with armed White individuals (*15*). Preliminary evidence suggests these events have repercussions even for individuals who are not personally involved. For example, adults who live in neighborhoods where a greater proportion of police interactions involve frisking or use of force are more likely to endorse psychological distress (*16*) and to report having diabetes, obesity, and hypertension (*17*, *18*).

Exposure to police violence in the neighborhood may be particularly salient for women. While women are less likely to be stopped by police themselves, women are more likely to experience gendered violence during police encounters, including sexual harassment and assault (*19*). In addition, starting from a young age, women are vicariously exposed through police stops of neighbors, family members, and friends (*20*). These incidents are likely to be stressful for women because they place family and friends at risk of mistreatment, violence, legal difficulties, and incarceration. Stress, in turn, can trigger behavioral and physiological changes that increase vulnerability to subsequent health problems. Even when stress is anticipatory in nature, e.g., concerns about the prospect of a loved one having a violent interaction with police, it can have repercussions for health (*21*–*26*). Consistent with these notions, studies have linked neighborhood exposure to fatal police violence with adverse reproductive outcomes, including a higher risk of PTD (*27*), a higher risk of delivering a low-birthweight infant (*28*), and a decline in the number of live births, suggestive of an increase in pregnancy loss (*29*).

We build on this evidence by considering the relationship of negative police interactions with PTD and CVD. As noted, there are pronounced, but not well-understood, racial disparities in PTD and CVD and mounting evidence to suggest that they share common pathologic mechanisms and may even represent different life course manifestations of the same underlying vascular abnormalities (*30*, *31*). We approximate negative police interactions based on formal complaints filed for excessive use of force by police (EFP complaints) and generate two distinct cohorts using electronic health records (EHR) from a single Chicago hospital to capture health over time. We had three hypotheses:

1) That incident PTD and CVD would be more common among women who live in neighborhoods with more excessive force complaints, even following adjustment for other forms of area-level social disadvantage, including neighborhood violence.

2) That these associations would be strongest for Black women, because compared with other racial/ethnic groups in Chicago, they are disproportionately exposed to violent police encounters.

3) That associations between exposure to excessive force complaints and PTD would persist in a maternal fixed-effects analysis. This analysis enhances control for unmeasured and mismeasured time-invariant confounders by comparing pregnancy outcomes within a woman (for women with multiple deliveries during the study period).

## RESULTS

### Pregnancy cohort

From the EHR-derived cohort of 119,010 live births to 89,711 women, 67,976 births to 53,478 women met inclusion criteria (fig. S1A). The mean age at delivery was 31.6 years (SD: 5.3; Table 1). The sample was 59.6% White, 20.2% Hispanic, 11.7% Black, and 8.5% Asian, and a majority were nulliparous (55.7%). A total of 8.1% of the sample delivered preterm (<37 weeks), and 7.7% delivered a small for gestational age (SGA) infant (birthweight <10th percentile for gestational age). A total of 15.8% of women had an EFP complaint filed in their block group in the year leading up to delivery, and 10.2% were exposed to a homicide during the same period (fig. S2). Compared with White women, Black women were twice as likely to be exposed to an EFP complaint (27.6% versus 13.9%) and to deliver preterm (14.3% versus 6.8%).

**Table 1.** Sample maternal characteristics for the pregnancy cohort stratified by exposure to police excessive force complaints in the block group (≥1) in the year leading up to delivery (*n* = 67,976 deliveries). BG, block group.

**Mean (SD) or *n* (%)**	**Total** **(*n* = 67,976)**	**Unexposed** **(*n* = 57,202)**	Exposed* **(*n* = 10,774)**
Age at delivery, years	31.6 (5.3)	31.7 (5.2)	30.9 (5.6)
Race/ethnicity			
Hispanic	13,726 (20.2)	11,794 (20.6)	1,932 (17.9)
Black (non- Hispanic)	7,974 (11.7)	5,771 (10.1)	2,203 (20.5)
White (non-Hispanic)	40,483 (59.6)	34,873 (61.0)	5,610 (52.1)
Asian (non-Hispanic)	5,793 (8.5)	4,764 (8.3)	1,029 (9.5)
Insurance at delivery			
Private	49,377 (72.7)	42,099 (73.6)	7,278 (67.6)
Public	18,012 (26.5)	14,656 (25.6)	3,356 (31.2)
Self-pay	565 (0.8)	430 (0.8)	135 (1.2)
Nulliparous	37,857 (55.7)	31,707 (55.4)	6,150 (57.1)
Composite SES of the block group^†^	0.0 (0.9)	0.0 (0.9)	−0.2 (0.9)
Median household income of block group	81,489 (38,880)	82,639 (39,095)	75,422 (37,145)
% in BG with some college or more	73.4 (23.0)	73.8 (22.8)	71.3 (23.7)
% in BG with income above poverty threshold	84.7 (12.7)	85.3 (12.2)	81.4 (14.5)
Homicide in block group in year before delivery	6,939 (10.2)	5,171 (9.0)	1,768 (16.4)
Violent crime rate (per 100 people)	0.8 (1.7)	0.6 (1.5)	1.3 (2.6)
PTD (<37 weeks)	5,504 (8.1)	4,500 (7.9)	1,004 (9.3)
Early PTD (<34 weeks)	2,010 (3.0)	1,611 (2.8)	399 (3.7)
SGA (<10th percentile)^‡^	5,259 (7.7)	4,328 (7.6)	931 (8.7)

*Considered exposed if at least one police excessive force complaint was filed in the block group in the year leading up to delivery.

†Mean of standardized median household income of the block group, percentage in the block group with some college or more, and percentage in the block group with income above the poverty threshold.

‡Birthweight <10th percentile for gestational age and sex (*52*).

Associations between exposure to an EFP complaint and risk of PTD were evaluated using Cox regression, stratified by race/ethnicity. Models were adjusted for age, parity, year of delivery, and block group SES, homicide, and population size. Exposure to an EFP complaint was associated with higher risk of PTD among Black women [hazard ratio (HR): 1.19; 95% confidence interval (CI): 1.04 to 1.35; Table 2; see table S1 for all regression estimates]. No associations were observed among other racial or ethnic groups.

**Table 2.** Associations between exposure to police excessive force complaints in the block group (≥1) in the year leading up to delivery and adverse outcomes in the pregnancy cohort, stratified by race/ethnicity (*n* = 67,976 deliveries). OR, odds ratio.

	**Hispanic** (*n* = 13,726)		**Black** (*n* = 7,974)		**White** (*n* = 40,483)		**Asian** (*n* = 5,793)	
	**HR**	**95% CI**	**HR**	**95% CI**	**HR**	**95% CI**	**HR**	**95% CI**
PTD (<37 weeks)*								
Unadjusted	0.93	0.78–1.11	1.29	1.13–1.46	1.08	0.96–1.21	0.93	0.71–1.22
Adjusted^†^	0.93	0.78–1.10	1.19	1.04–1.35	1.04	0.93–1.16	0.91	0.69–1.20
Early PTD (<34 weeks)*								
Unadjusted	0.98	0.76–1.27	1.29	1.08–1.54	1.05	0.85–1.29	1.15	0.77–1.70
Adjusted^†^	1.00	0.77–1.30	1.20	1.00–1.45	1.04	0.84–1.29	1.12	0.75–1.68
**SGA (<10th percentile)^‡^**								
	OR	95% CI	OR	95% CI	OR	95% CI	OR	95% CI
Unadjusted	0.92	0.76–1.10	1.14	0.99–1.31	1.03	0.92–1.16	1.17	0.95–1.44
Adjusted^†^	0.90	0.75–1.08	1.16	1.01–1.35	1.05	0.93–1.18	1.15	0.93–1.42

*HRs from Cox regression.

†Adjusted for age at delivery, parity, year of delivery, and block group SES, homicide frequency, and population size.

‡Odds ratios from generalized estimating equations; SGA is defined as birthweight <10th percentile for gestational age and sex (*52*).

To determine whether the risks were different across racial and ethnic groups, we used a full interaction model where EFP exposure and all covariates were interacted with race/ethnicity. In this model, which yields parameter estimates equivalent to those obtained from the stratified models, the *P* value for interaction between exposure and race/ethnicity was 0.09. As this maximally flexible model may be less precise (*32*), we also used an alternative interaction model using backward elimination to retain statistically significant interaction terms. Using this approach, the *P* value for interaction between exposure and race/ethnicity was 0.03 (table S2).

The point estimate for Black women was consistent in a sensitivity analysis where PTD was defined as <34 weeks (HR: 1.20; 95% CI: 1.00 to 1.45; Table 2) and in a sensitivity analysis adjusting for neighborhood violent crime rate rather than homicide exposure (HR: 1.20; 95% CI: 1.05 to 1.36; table S3). They were also consistent when two additional conservative approaches to model estimation were used: first, where exposure was modeled as a time-dependent variable (HR: 1.12; 95% CI: 0.97 to 1.29; table S4), and second, in a block group fixed-effects analysis (HR: 1.18; 95% CI: 1.02 to 1.38; table S5).

In the maternal fixed-effects analysis of 12,986 women with multiple deliveries during the study period, associations were similar among Black women. The exposed pregnancy was 1.28 times as likely to deliver preterm (95% CI: 0.87 to 1.90), although CIs were wide because of the small sample size (*n* = 113 Black women with ≥1 PTD and exposure discordance; table S6).

**Fig. 1.**
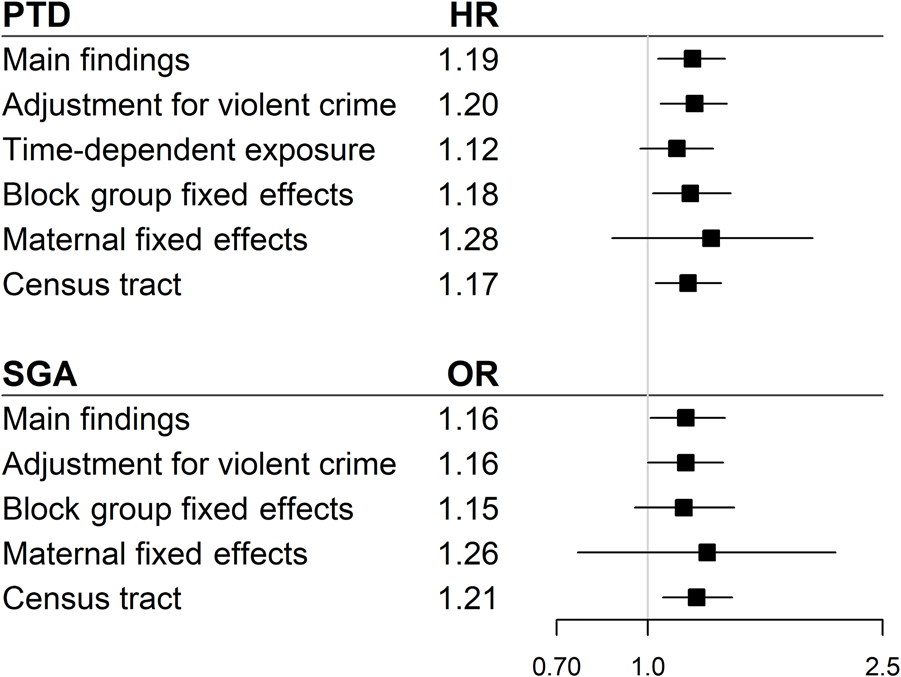
Summary plot of associations between EFP exposure and pregnancy outcomes among Black women. Plot displays adjusted estimates and 95% CIs.

As an additional sensitivity analysis, we evaluated associations at the census tract, which may provide improved control for area-based measures of SES and additionally allows for control of a measure of neighborhood stability (proportion of residents who lived in the same home the year prior), which is not available at the block group level. Results were consistent for exposure in the census tract; exposed Black women were 1.17 times as likely to deliver preterm (<37 weeks’ gestation; 95% CI: 1.03 to 1.33; table S7) and 1.22 times as likely to deliver early preterm (<34 weeks’ gestation; 95% CI: 1.02 to 1.46). To facilitate comparison of results from different modeling strategies, adjusted associations for main findings and sensitivity analyses among Black women are presented in Fig. 1.

Independent of PTD, Black women have increased risk of delivering infants who are SGA, defined as birthweight <10th percentile based on the newborn’s sex and gestational age. SGA infants are at increased risk of neonatal morbidity and mortality and have worse cognitive outcomes in childhood (*33*, *34*). Thus, in a secondary analysis, we used generalized estimating equations to assess associations between exposure to EFP complaints and SGA infant. After adjustment for covariates, exposed Black women were 1.16 times as likely to deliver an SGA infant as compared with unexposed Black women (95% CI: 1.01 to 1.35; Table 2). Exposure was not associated with delivery of an SGA infant among any other racial or ethnic group, although in an equivalent interaction model, the *P* value for interaction between EFP exposure and race/ethnicity was 0.13. Using a backward elimination approach to retain statistically significant interaction between race/ethnicity and covariates, the *P* value for interaction between exposure and race/ethnicity was 0.09 (table S2).

Results for SGA infant were consistent when EFP exposure and covariates were defined on the basis of the census tract, with elevated odds of SGA infant observed among Black women [odds ratio (OR): 1.21; 95% CI: 1.06 to 1.39; table S7]. Results were also consistent when neighborhood crime was measured on the basis of violent crime rate rather than homicide exposure (OR: 1.16; 95% CI: 1.00 to 1.34; table S3). Point estimates were also similar in the block group fixed-effects analysis (OR: 1.15; 95% CI: 0.95 to 1.40; table S5) and the maternal fixed-effects analysis (OR: 1.26; 95% CI: 0.76 to 2.08; table S6).

### CVD cohort

In the CVD sample, the EHR-derived cohort included 15,352 women, 6773 of whom met inclusion criteria (fig. S1B). Among women in the analytic CVD cohort, 60.1% were White and 39.9% were Black (Table 3). At the time of the index visit, 61.7% were overweight or obese based on body mass index (BMI), and 7.5% had been diagnosed with diabetes and 34.8% with hypertension. Over a median follow-up of 8.0 years (interquartile range: 4.7 to 11.9), 14.2% were diagnosed with incident CVD. Black women were more likely to be in the highest decile of exposure to EFP complaints (≥0.56 EFP complaints per year; 16.2% versus 7.1%; fig. S3) and to develop incident CVD (17.3% versus 12.1%) as compared with White women.

**Table 3.** Sample characteristics for women in the CVD cohort, by exposure to police excessive force complaints in the block group (top 10th percentile of exposure distribution) (*n* = 6773).

***n* (%) or mean (SD)**	**Total** (*n* = 6773)	**Unexposed** (*n* = 6046)	Exposed* (*n* = 727)
Age at index visit	49.6 (12.3)	49.7 (12.4)	48.8 (12.1)
Race/ethnicity			
Black (non-Hispanic)	2,704 (39.9)	2,265 (37.5)	439 (60.4)
White (non-Hispanic)	4,069 (60.1)	3,781 (62.5)	288 (39.6)
Insurance			
Private	3,684 (54.4)	3,319 (54.9)	365 (50.2)
Medicaid	265 (3.9)	215 (3.6)	50 (6.9)
Medicare	2,359 (34.8)	2,107 (34.8)	252 (34.7)
Self-pay	465 (6.9)	405 (6.7)	60 (8.2)
Smoking status (index visit)			
Never smoker	4,550 (67.2)	4,044 (66.9)	506 (69.6)
Former smoker	669 (9.9)	586 (9.7)	83 (11.4)
Current smoker	1,554 (22.9)	1,416 (23.4)	138 (19.0)
BMI category (index visit)			
Normal weight or underweight (<25 kg/m^2^)	2,593 (38.3)	2,362 (39.1)	231 (31.8)
Overweight (25 to 30 kg/m^2^)	1,776 (26.2)	1,601 (26.5)	175 (24.1)
Obese (≥30 kg/m^2^)	2,404 (35.5)	2,083 (34.4)	321 (44.1)
Diabetes at index visit	511 (7.5)	421 (7.0)	90 (12.4)
Hypertension at index visit	2,359 (34.8)	2,065 (34.2)	294 (40.4)
Composite SES of BG^†^	0.0 (0.9)	0.0 (0.8)	−0.3 (1.0)
Median household income of BG	72,109 (36,444)	72,715 (36,386)	67,106 (36,563)
% in BG with some college or more	73.5 (20.9)	74.3 (20.3)	66.8 (24.0)
% in BG with income above poverty threshold	83.6 (13.8)	84.3 (13.2)	77.4 (16.8)
Homicide frequency (top decile of homicides per year)	731 (10.8)	517 (8.6)	214 (29.4)
Violent crime rate (per 100 people per year)	0.2 (0.5)	0.2 (0.5)	0.4 (0.6)
Incident CVD^‡^	961 (14.2)	819 (13.6)	142 (19.5)

*Considered exposed if in the top decile for average number of police excessive force complaints per year in the block group during the follow-up period (≥0.56 complaints per year of follow-up).

†Mean of standardized median household income of the block group, percentage in the block group with some college or more, and percentage in the block group with income above the poverty threshold.

‡Includes ischemic heart disease, cerebrovascular disease, peripheral vascular disorders, and congestive heart failure.

We used Cox regression models stratified by race to evaluate associations between EFP exposure and development of incident CVD. Models were adjusted for year of index visit and block group SES, homicide, and population size. Black women in the highest decile of EFP complaints were 1.47 times as likely to develop incident CVD as compared with Black women with lower exposure (95% CI: 1.16 to 1.86; Table 4). The association persisted after controlling for traditional CVD risk factors at the index visit, including smoking status, BMI, diabetes, and hypertension (HR: 1.42; 95% CI: 1.12 to 1.79; see table S8 for all regression estimates) and in a model controlling for violent crime rate rather than homicide exposure (HR: 1.40; 95% CI: 1.11 to 1.77; table S9). The association was also consistent in a sensitivity analysis restricted to those with at least 5 years of follow-up (HR: 1.44; 95% CI: 1.04 to 1.99), further implying that results were not confounded by subclinical disease at baseline (table S10). EFP complaints were not significantly associated with CVD among White women; the HR was 1.28 (95% CI: 0.92 to 1.77; Table 4). Using an equivalent interaction model, the *P* value for interaction between EFP exposure and race/ethnicity was 0.55.

**Table 4.** Associations between exposure to police excessive force complaints in the block group (top decile) and incident CVD among women in the CVD cohort, stratified by race (*n* = 6773).

	**Black** (*n* = 2704)		**White** (*n* = 4069)	
	**HR**	**95% CI**	**HR**	**95% CI**
Model 1^*^	1.64	1.30–2.06	1.29	0.92–1.80
Model 2^†^	1.47	1.16–1.86	1.29	0.91–1.82
Model 3^‡^	1.42	1.12–1.79	1.28	0.92–1.77

*Unadjusted.

†Adjusted for year of index visit and block group SES, homicide frequency, and population size.

‡Additionally adjusted for smoking status, BMI, hypertension, and diabetes, all at the index visit.

Because of the strong right skew of the EFP distribution, we could not perform a dose-response analysis, asking whether each increment in exposure conferred additional CVD risk. As an alternative, we compared each of the top three deciles of EFP exposure to the bottom 70% of the distribution (all averaged <0.22 EFP complaints per year). There was a significant linear trend (*P* < 0.01), with CVD risk increasing progressively from the 8th to 9th to 10th decile of EFP exposure. However, only exposure in the 10th decile was significantly associated with CVD risk among Black women (HR: 1.47; 95% CI: 1.14 to 1.90; table S11).

**Fig. 2.**
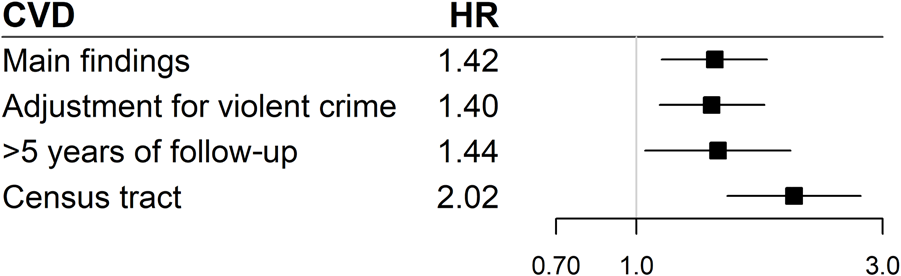
Summary plot of associations between EFP exposure and incident CVD among Black women. Plot displays adjusted estimates and 95% CIs.

As with PTD, we conducted a sensitivity analysis based on census tract estimates of exposure and covariates. Black women in the highest decile of EFP complaints (≥1.57 EFP complaints per year) were 2.02 times as likely to develop incident CVD (95% CI: 1.50 to 2.72; table S12). Associations were elevated but not statistically significant among White women (HR: 1.56; 95% CI: 0.82 to 2.96). We also present adjusted associations among Black women in Fig. 2 to facilitate comparison of estimates from the main analysis, sensitivity analysis restricted to those with at least 5 years of follow-up, sensitivity analysis controlling for violent crime, and sensitivity analysis based on the census tract.

In studies of neighborhood exposures, spatial correlations between covariates can result in a violation of the positivity assumption, which requires variation in exposure within strata of confounders (*35*, *36*). Correlations between exposure to EFP complaints and covariates, such as race/ethnicity, neighborhood SES, and homicide, may result in subgroups that always or never experience exposure. When this occurs, results for these groups must be extrapolated. To determine whether this was the case in our cohorts, we calculated propensity scores for exposure to evaluate violations of the positivity assumption (*36*). In both the PTD and CVD cohorts, the exposed and unexposed exhibited similar propensity score distributions and ranges (fig. S4), indicating that violations of the positivity assumption are unlikely to be a concern here.

## DISCUSSION

These results indicate that in neighborhoods where residents frequently complain about the police using excessive force, Black women have a 19% greater risk of PTD, 16% greater risk of delivering an SGA infant, and a 42% greater risk of CVD, even after accounting for the neighborhood’s socioeconomic conditions and homicide frequency. In comparison, smoking, an established risk factor for both PTD and CVD, is associated with a nearly 30% greater risk of PTD and 70% greater risk of CVD (*37*–*39*). Although these findings must be interpreted with caution until replicated and substantiated, they add to growing evidence suggesting that police violence adversely affects the health of Black communities (*16*–*18*, *27*, *29*, *40*–*42*). Our study extends these previous reports by using two large samples, individual-level diagnoses made by physicians, and, in the pregnancy cohort, a fixed-effects analysis that minimizes the potential for confounding by stable maternal characteristics.

One obvious question that arises from these observations is what mechanism(s) could plausibly be involved. The EHR datasets do not contain indicators needed to address this question effectively. However, if the findings reflect a causal phenomenon, there are plausible scenarios to consider in future research. Neighborhood violence is related to adverse pregnancy and cardiovascular outcomes, presumably via stress-evoked changes in lifestyle and physiology (*43*–*45*). These same pathways may be involved in any health consequences of police violence. Studies of civilian killings by police and neighborhood violence more generally indicate that these stressors affect not only those who are directly involved but also members of the broader community. These “indirect” effects can manifest in unfavorable changes in sleep patterns, mental health, diurnal cortisol release, low-grade inflammation, and cardiometabolic risk (*21*–*24*). These pathways have been tied to PTD, SGA, and CVD and could explain the observations here. Future research should clarify what role, if any, these candidate mechanisms play. Since adverse pregnancy outcomes and CVDs share a number of common pathogenic mechanisms, it would make sense to prioritize consideration of these pathways, e.g., persistent inflammation and vascular dysfunction (*30*).

Another question that these findings raise is why the health risks associated with EFP complaints were most pronounced among Black women. In Chicago, Black residents disproportionately experience use of force by police. While about one-third of Chicago residents are Black, three-quarters of reported instances of police use of force involve a Black resident (*14*). This pattern is echoed in our data, where 69% of EFP complaints were filed by Black residents (*46*) and Black women disproportionately live in high-exposure neighborhoods. These trends, along with the highly visible and public deaths of Black men in police custody, may render community incidents especially threatening to Black women.

There are several limitations of this study. We used complaints to define police violence, rather than patients’ reports of interactions with police, or police behavior in their neighborhoods. Additional data of this nature would help differentiate between the effects of personal and vicarious exposure to police violence. Furthermore, there are likely many determinants of whether a complaint is reported, including the nature of the incident, the person’s knowledge of the complaint process and willingness and ability to file a complaint, and the perception that filing a complaint is impactful. These determinants may vary by neighborhood, with some neighborhoods having a greater propensity for filing complaints. If marginalized communities are less likely to file reports, then their exposure to EFP may be underestimated here. As these communities are often at greater risk of adverse health outcomes, this may result in differential exposure misclassification, and our results may be biased toward the null, i.e., underestimate the population effect size. Similarly, our analysis only considers EFP complaints occurring near the home, which does not capture all situations where a person may be exposed to police violence.

We also lack data on the total number of police interactions in each neighborhood, which would clarify what proportion involved excessive force. More police interactions could increase the likelihood that a complaint is filed. If so, our EFP variable could reflect racial bias in total number of encounters rather than racial bias in using excessive force. However, our models adjust for measures of block group SES, crime, and population size, which should presumably capture variation in the number of police interactions. Similarly, there may be other neighborhood-based confounders that are not accounted for. However, results were consistent in the block group fixed-effects models, which control for time-invariant block group characteristics (both unmeasured and unknown). Consistency of the findings suggests that confounding by neighborhood characteristics (to the extent they are stable over time) may not explain the observed associations.

Our study is also limited by the data available in EHRs. For example, we were unable to separate spontaneous and iatrogenic PTD or thoroughly track changes in where patients lived over time. The pregnancy cohort was also restricted to live births. Since police violence is associated with pregnancy loss (*29*), there is potential here for exposure-induced selection. This may bias results either through conditioning on a collider (live births) or a depletion of fetuses susceptible to PTD (*47*, *48*). Again, in both scenarios, our results would likely underestimate the association between exposure to EFP complaints and PTD. A similar selection mechanism may affect the CVD cohort, as neighborhood policing practices may negatively affect health care utilization (*49*), which is required for selection into the cohort. Last, the data are from a single institution in Chicago and may not be representative of the city of Chicago or generalizable to other locations; future studies are needed to determine how they generalize. However, the city of Chicago has the second-largest municipal police department in the United States and has the widest life expectancy gap of any major metropolitan area in the United States, making this an opportune location to investigate relationships between police violence and health outcomes (*50*).

Despite these limitations, this study highlights police violence as a potential contributor to PTD and CVD risk in Black women. There are persistent racial disparities in these outcomes in the United States, which continue to be poorly understood, because they cannot be explained by traditional risk factors and widen as women’s socioeconomic conditions improve. Furthermore, exposure to EFP may be similar across levels of SES for Black women, as affluent Black families tend to live in neighborhoods with lower SES and higher rates of crime as compared with similarly affluent White families (*12*). Differential exposure to excessive police force could be an overlooked contextual exposure that contributes to these disparities. Future research should consider these possibilities, survey women’s direct experiences with law enforcement and perceptions of police behavior in their community, and attempt to elucidate underlying mechanisms for the pattern seen here.

## MATERIALS AND METHODS

### Study samples

The analyses involved two distinct cohorts derived from EHRs. In the pregnancy cohort, records were obtained from all singleton live births at a single Chicago hospital between March 2008 and March 2018 (*n* = 119,010 births to 89,711 women). Those living outside Chicago and with missing or incomplete addresses were excluded (fig. S1A). Women with estimated dates of conception more than 20 weeks before the start of the study period or within 44 weeks of the end of the study period were excluded to account for fixed cohort bias (*51*). In addition, women identified as “other” or missing race/ethnicity were excluded. The final sample included 67,976 births to 53,478 women.

In the CVD cohort, records were obtained for patients between the ages of 30 and 80 who visited an internal medicine physician at a single Chicago hospital between January 2001 and December 2018. The EHR review was limited to those who were CVD free at the index visit (defined as a history of ischemic heart disease, cerebrovascular disease, peripheral vascular disorders, or congestive heart failure) and who had a follow-up visit at least 2 years later (*n* = 15,352). Patients living outside Chicago or with missing or incomplete addresses were excluded (fig. S1B), as were those missing race/ethnicity or listed as other. Patients listed as Hispanic or Asian were also excluded because of small sample sizes. We also excluded patients who developed CVD within 2 years of the index visit because they likely had considerable preclinical disease at baseline. After exclusions, the analytic sample included 6773 women.

### Exposure

Data on complaints about police conduct were obtained from the Invisible Institute’s Citizens Police Data Project (*46*). Using a Freedom of Information Act (FOIA) request, the Invisible Institute acquired data on formal complaints filed against Chicago police through June 2018. Our analysis is limited to complaints filed for use of force by police. During the time frame of the pregnancy and CVD cohorts, there were 6798 and 13,224 unique incidents where use of force complaints were filed, respectively (fig. S2). Location data were available for 6003 complaints in the pregnancy cohort (88.3%) and 11,815 complaints in the CVD cohort (89.3%). Location of the incident was geocoded using ArcGIS Pro 2.5 at the block group level of resolution, a geographic unit from the Census Bureau, which typically has 600 to 3000 residents. Women’s home addresses were geocoded in the same fashion on the basis of hospital billing data at the time of delivery (pregnancy cohort) or index visit (CVD cohort). As a majority of use of force complaints are described as excessive force (76%), we use the term “excessive force by police” or EFP, to describe exposure [other common use of force descriptors include unnecessary physical contact (11%) and unnecessary display of a weapon (7%)].

In the pregnancy cohort, patients were considered exposed if at least one EFP complaint was filed in their block group in the year leading up to delivery. We used a 1-year period to ensure an equal exposure period for all patients, regardless of gestation length. In the CVD cohort, we calculated the average number of complaints in a patient’s block group per year since there is not a clearly defined exposure period, patients have varying lengths of follow-up, and CVD generally develops over the course of decades. Follow-up length was determined as the time between the index visit and either the diagnosis date or, for those without the outcome, the most recent visit. For example, a patient exposed to two complaints in 3 years of follow-up would have 0.67 complaints per year.

By calculating exposure as the average number of complaints per year of follow-up, an artificial relationship was created between exposure and length of follow-up. For example, for a patient to have an exposure of 0.1 complaints per year, they must have a minimum of 10 years of follow-up. Patients with less follow-up time cannot have a similarly low exposure [e.g., a patient with 2 years of follow-up can have an exposure of 0 or ≥0.5 (one complaint in 2 years), but they cannot have an exposure between 0 and 0.5]. This dependence yields misleading results, particularly for those with low, but nonzero, exposure, where they must have a long period of follow-up time and are more likely to be censored than to experience the outcome. To alleviate dependence on length of follow-up and because of the zero-inflated and right-skewed distribution (fig. S3), exposure in the CVD cohort was dichotomized at the top decile (0.56 EFP complaints per year). As a sensitivity analysis, we compared the top three deciles of exposure to the bottom 70% of the data to allow for a nonlinear relationship and to investigate a dose-response relationship. Groups were defined as follows: <70th percentile, <0.22 EFP complaints per year; 70th to 80th percentile, 0.22 to 0.32 EFP complaints per year; 80th to 90th percentile, 0.32 to 0.56 EFP complaints per year; and ≥90th percentile, ≥0.56 EFP complaints per year.

### Outcomes

In the pregnancy cohort, the primary outcome was PTD, defined as delivery <37 weeks of gestation. We also evaluated early PTD on the basis of gestational age <34 weeks. The secondary outcome was SGA infant, defined as birthweight <10th percentile for gestational age and sex (*52*). In the CVD cohort, the outcome of incident CVD included a composite of four diagnoses: ischemic heart disease, cerebrovascular disease, peripheral vascular disorders, and congestive heart failure [based on *International Classification of Diseases* (*ICD*) codes; table S13].

### Covariates

Covariates in both cohorts were selected a priori on the basis of associations reported in the literature and patterning by race/ethnicity. In the pregnancy cohort, covariates included age at delivery, race/ethnicity (Hispanic, Black, White, or Asian, based on EHR), and parity. In the CVD cohort, covariates from the index visit included age, race (Black or White, based on EHR), BMI, smoking status, and diabetes and hypertension diagnoses (based on *ICD* codes; table S13). To account for temporal trends, year of delivery (pregnancy cohort) and year of index visit (CVD cohort) were also included as covariates.

Neighborhoods with high rates of EFP complaints may experience other forms of social disadvantage relevant to health. Therefore, we also included covariates that reflected the block group’s SES and violent crime rate. Using the 5-year American Community Survey (ACS) estimates, SES was calculated by averaging standardized measures of block group median income, percentage of the block group who have completed some college or more, and percentage of the block group with a household income at or above the poverty threshold (income-to-poverty ratio ≥ 1). We also included the population size of the block group. The mid-point of the 5-year estimates was matched to the delivery year in the pregnancy cohort and index visit year in the CVD cohort (e.g., a woman with either a delivery or an index visit in 2013 was assigned estimates from the 2011–2015 ACS).

In the main analyses, we estimated violent crime based on homicides in the block group because homicides are less likely to be underreported as compared with other serious violent crimes, such as rape, sexual assault, robbery, and aggravated assault, of which an estimated 42% are not reported to police (*53*). Furthermore, crime reporting may be dependent on police presence in the neighborhood (*54*). In the pregnancy cohort, we captured whether ≥1 homicide occurred in the year leading up to delivery. In the CVD cohort, the average number of homicides per year over follow-up was calculated and then dichotomized at the top decile. In a sensitivity analysis, we substituted a broader definition of violent crime to capture variation in crime exposure. For this analysis, violent crimes were defined on the basis of the Chicago Police Department’s violent crime index and included homicide, criminal sexual assault, robbery, aggravated assault, and aggravated battery (*55*). In both cohorts, we calculated the total number of violent crimes occurring in the block group during the period of interest (year leading up to delivery in the pregnancy cohort or follow-up time in the CVD cohort). Violent crime exposure was then summarized as the average rate per 100 residents per year. In the CVD cohort, crime was categorized on the basis of quartiles to alleviate the dependence on follow-up time. Crime data were obtained from Chicago’s Data Portal.

### Statistical analysis

In the pregnancy cohort, associations were estimated using Cox regression to account for the time-dependent nature of PTD. Gestational age was used as the time scale with adjustment for left truncation (gestational age of earliest delivery; see eq. S1). Variances were estimated using methods to account for non-nested clustering among those in the same block group and those with multiple deliveries (*56*–*58*). Observations were censored at 37 or 34 weeks, depending on the outcome. We also conducted a secondary analysis using extended Cox regression with a time-dependent variable for EFP complaints. In contrast to the main analysis, the time-dependent variable is a more conservative approach that considers patients unexposed until a complaint occurs in their block group, after which they are considered exposed. The time-dependent EFP variable was also structured to only capture exposure during pregnancy. For models examining SGA infant, we used generalized estimating equations with methods to account for non-nested clustering (*57*). All models were stratified by race/ethnicity, although we also tested whether the effect of exposure differed by race/ethnicity using interaction models. Models were adjusted for age (<20, 20 to 35, and >35), year of delivery, parity (nulliparous), and block group SES, homicide frequency, and population size.

Although covariate adjustment can reduce the potential for confounding, it does not eliminate it because some potential confounds are not measured or mismeasured. To address this problem, we conducted two fixed-effects analyses. In the first, we analyzed the subset of women who had multiple deliveries during the study period (*n* = 12,986). This approach minimizes the influence of all stable maternal characteristics by comparing pregnancies for the same woman. In the second, we compared women living in the same block groups to control for stable block group characteristics. Associations were estimated using stratified Cox regression (PTD) or conditional logistic regression (SGA) (*56*).

In the CVD cohort, associations were estimated using Cox regression with a robust sandwich estimator to account for clustering by block group (*56*). Age was used as the time scale, with adjustment for left truncation (age at index visit) and stratification by 5-year birthdate cohort (see eq. S2). Model 1 is unadjusted; model 2 is adjusted for SES, homicide frequency, and population size of the block group and year of index visit; and model 3 is additionally adjusted for baseline CVD risk factors: smoking status (never smoker, current smoker, and former smoker), BMI (<25, 25 to 30, and >30 kg/m^2^), diabetes, and hypertension at the index visit. As a sensitivity analysis, models were restricted to those with at least 5 years of follow-up before CVD diagnosis. A second sensitivity analysis allowed for a nonlinear relationship and investigated a dose-response relationship by comparing each of the top three deciles of exposure to the bottom 70%. All models were stratified by race to allow for flexibility in covariate estimation, although whether the association between exposure and CVD differed by race/ethnicity was formally tested using interaction models.

While we chose to define neighborhoods on the basis of block groups because the smaller area may increase the likelihood that an individual is aware of an incident resulting in a formal complaint, as a sensitivity analysis, we replicated the main analyses using census tract–level measures of exposure and neighborhood characteristics. Defining neighborhoods based on census tracts may also improve control of confounding by SES, as estimates from larger areas may be more stable (*59*). Models additionally controlled for the percentage of people who lived in the same house the prior year as a measure of neighborhood stability, which is not available for block groups.

In both studies, we used hot deck imputation to impute missing covariate data (*60*), and propensity scores to evaluate the positivity assumption and explore whether there was sufficient variability in exposure within strata of confounders (*35*, *36*). Proportional hazard assumptions were assessed using Schoenfeld residuals and time-dependent covariates. Analyses were performed using SAS version 9.4 (SAS Institute Inc., Cary, NC). Both studies were approved by the Northwestern University Institutional Review Board.

## Supplementary Materials

### This PDF file includes:

Figs. S1 to S4 Tables S1 to S13 Supplementary Equations
