# Supplementary material for: Complaints about excessive use of police force in women’s neighborhoods and subsequent perinatal and cardiovascular health
Source: Sci Adv. 2022 Jan 19;8(3):eabl5417. doi: 10.1126/sciadv.abl5417 (PMC8769548; doi:10.1126/sciadv.abl5417)
Supplement: Supplementary file 1 — Figs. S1 to S4 Tables S1 to S13 Supplementary Equations [file sciadv.abl5417_sm.pdf]

Science Advances
AAAS

Supplementary Materials for

Complaints about excessive use of police force in women's neighborhoods and subsequent perinatal and cardiovascular health

Alexa A. Freedman*, Andrew V. Papachristos, Britney P. Smart, Lauren S. Keenan-Devlin, Sadiya S. Khan, Ann Borders, Kiarri N. Kershaw, Gregory E. Miller

*Corresponding author. Email: alexa.freedman@northwestern.edu

Published 19 January 2022, *Sci. Adv.* **8**, eabl5417 (2022)
DOI: 10.1126/sciadv.abl5417

This PDF file includes:

Figs. S1 to S4
Tables S1 to S13
Supplementary Equations

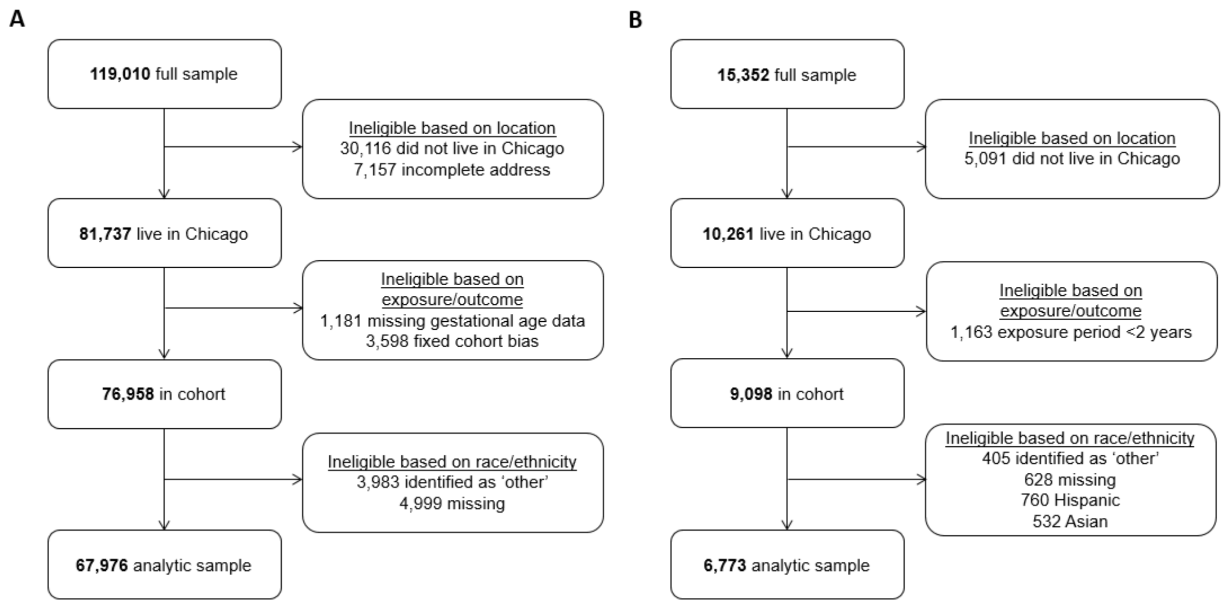

Fig. S1. Study inclusion for the pregnancy cohort (A) and the CVD cohort (B).

A B

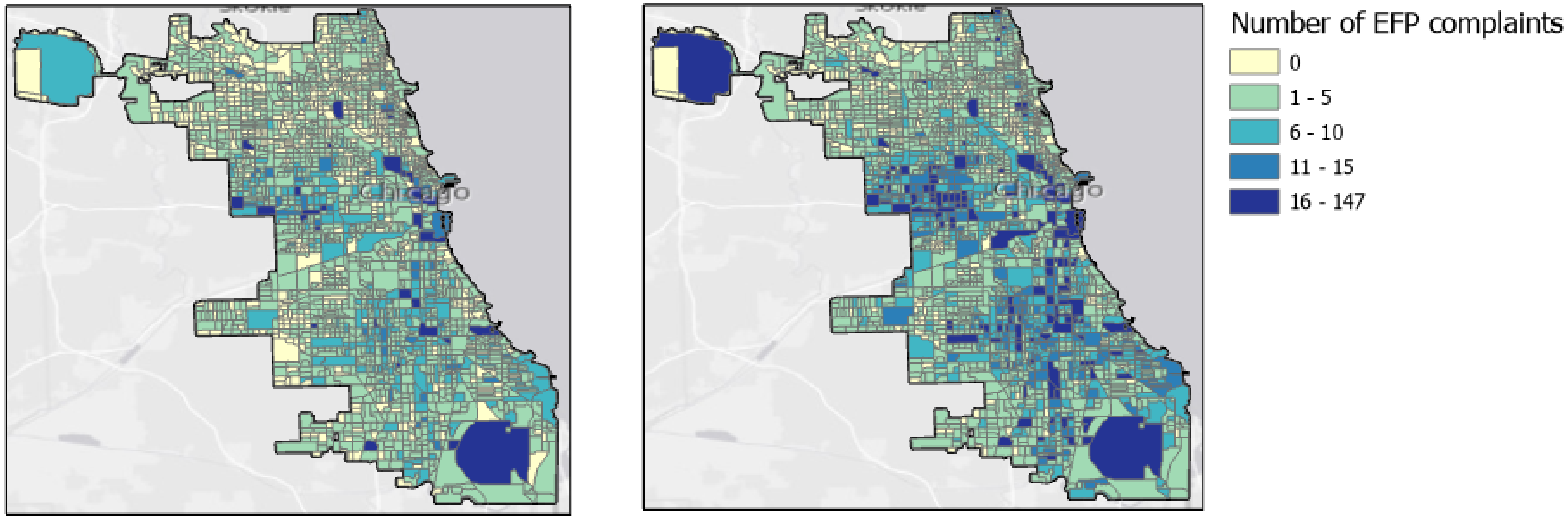

Fig. S2. Spatial distribution of complaints about excessive use of force by police (EFP complaints) during the period of (A) the pregnancy cohort (March 2007 – March 2018) and (B) the CVD cohort (January 2001 – June 2018). During the periods of the pregnancy and CVD cohorts, there were 6,003 and 11,815 EFP complaints with location data, respectively.

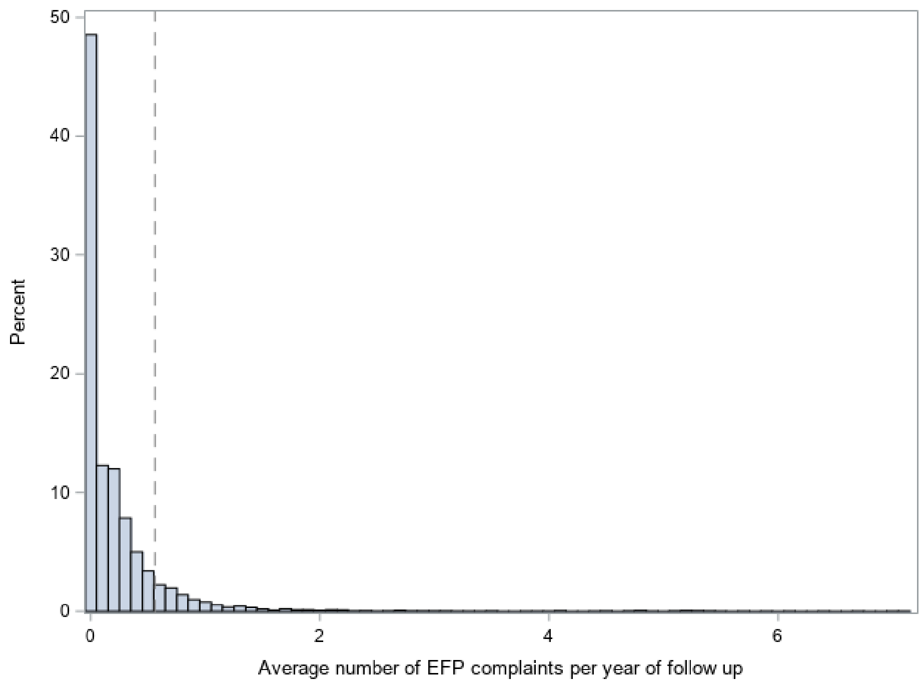

Fig. S3. Distribution of exposure to excessive use of force by police (EFP) complaints in the CVD cohort. Exposure was defined as the average number of EFP complaints in the block group per year of follow up and ranged from 0 to 7.05 with a median of 0.07. Exposure was dichotomized at the top decile (≥0.56 EFP complaints per year), indicated by the dashed line.

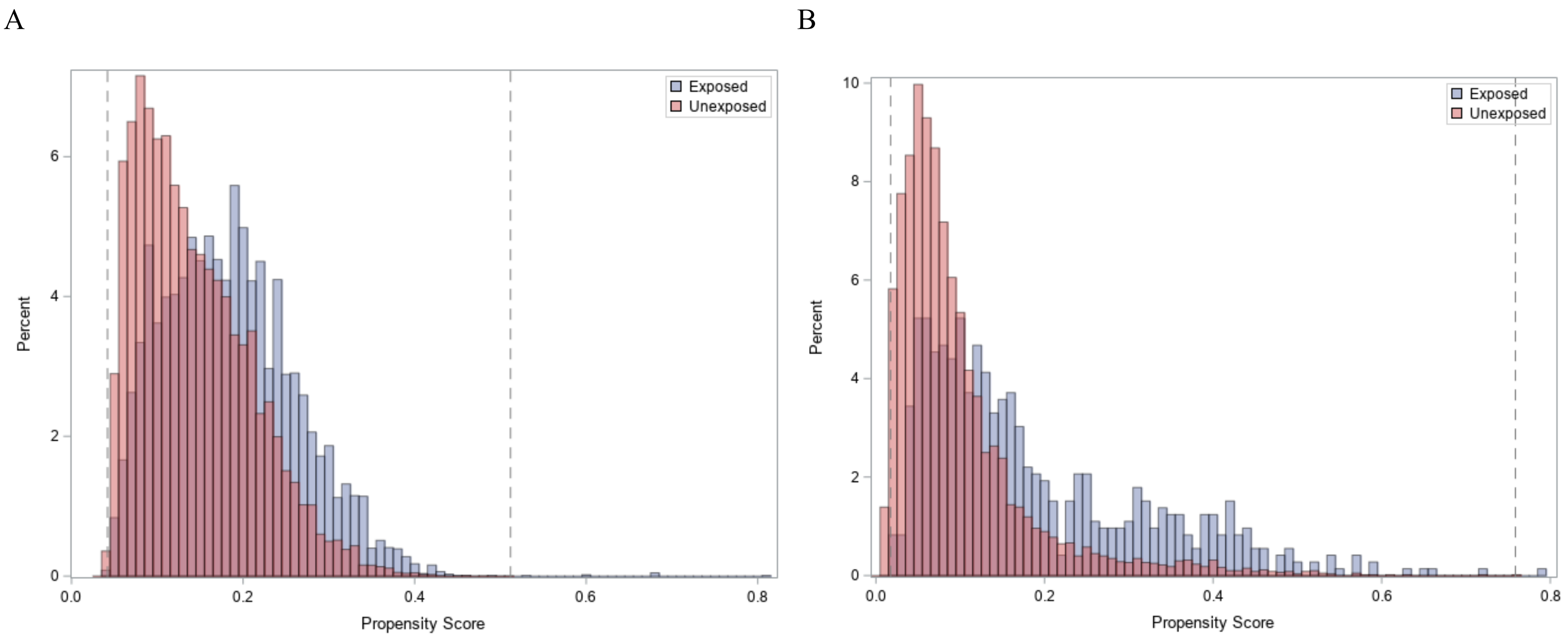

Fig. S4. Histograms of propensity scores for individuals exposed and unexposed to complaints about excessive use of force by police in the pregnancy cohort (A) and the CVD cohort (B). In the pregnancy cohort, exposure to complaints about excessive use of force by police was modeled as a function of race/ethnicity, age at delivery, parity, year of delivery, and block group socioeconomic status, homicide exposure, and population size. In the CVD cohort, exposure was modeled as a function of index visit measures of race, age, smoking status, body mass index, hypertension, and diabetes, year of index visit, and block group socioeconomic status, homicide exposure, and population size. The dashed lines indicate the area of common support, or the range in which the propensity scores for the exposed and unexposed overlap. Excluding those outside the area of common support would result in the exclusion of 71 (0.1%) patients in the pregnancy cohort and 147 (2.2%) patients in the CVD cohort.

Table S1. Parameter estimates for adjusted model of associations between exposure to police excessive force complaints in the block group (≥1) in the year leading up to delivery and preterm delivery (<37 weeks) in the pregnancy cohort using Cox regression, stratified by race/ethnicity (n=67,976 deliveries).

	**Hispanic** (n=13,726)		**Black** (n=7,974)		**White** (n=40,483)		**Asian** (n=5,793)	
	β	95% CI	β	95% CI	β	95% CI	β	95% CI
EFP complaint	-0.07	-0.25, 0.10	0.17	0.04, 0.30	0.04	-0.08, 0.15	-0.09	-0.37, 0.18
Homicide	-0.03	-0.20, 0.13	0.11	-0.03, 0.24	0.04	-0.12, 0.19	0.26	-0.13, 0.65
Age, <20 years*	0.22	0.02, 0.43	0.34	0.12, 0.56	0.38	-0.34, 1.11	-10.97	-11.73, -10.20
Age, >35 years*	0.20	0.04, 0.35	0.14	-0.04, 0.31	0.16	0.07, 0.25	0.35	0.11, 0.59
Nulliparous	0.00	-0.12, 0.13	-0.17	-0.29, -0.05	0.27	0.19, 0.35	0.16	-0.06, 0.37
BG SES	-0.05	-0.13, 0.03	-0.13	-0.21, -0.05	-0.04	-0.10, 0.02	-0.04	-0.18, 0.10
BG population	-0.07	-0.15, 0.01	0.01	-0.07, 0.09	0.04	0.00, 0.08	0.00	-0.07, 0.06
Delivery year	-0.01	-0.03, 0.01	-0.02	-0.04, 0.00	-0.01	-0.02, 0.00	-0.02	-0.06, 0.01

Abbreviations: CI – confidence interval; EFP complaint – complaint about excessive force by police; BG SES – block group socioeconomic status; BG population – block group total population (per 1,000)

* Reference group for age: 20-35 years

Table S2. Associations between exposure to police excessive force complaints in the block group (≥1) in the year leading up to delivery and adverse outcomes in the pregnancy cohort using interaction models (n=67,976 deliveries).

	**Hispanic** (n=13,726)		**Black** (n=7,974)		**White** (n=40,483)		**Asian** (n=5,793)		**Interaction p-value**
	HR	95% CI	HR	95% CI	HR	95% CI	HR	95% CI	
**Preterm delivery (<37 weeks)** *									
Unadjusted	0.90	0.76, 1.08	1.24	1.09, 1.41	1.05	0.94, 1.17	0.92	0.70, 1.20	0.02
Adjusted†	0.91	0.76, 1.08	1.22	1.08, 1.39	1.04	0.93, 1.17	0.92	0.70, 1.21	0.03
**SGA infant**‡	OR	95% CI	OR	95% CI	OR	95% CI	OR	95% CI	
Unadjusted	0.91	0.75, 1.09	1.18	1.03, 1.36	1.03	0.92, 1.16	1.17	0.95, 1.44	0.09
Adjusted§	0.91	0.75, 1.08	1.18	1.02, 1.35	1.04	0.92, 1.16	1.16	0.94, 1.43	0.09

Abbreviations: CI – confidence interval; HR – hazard ratio; OR – odds ratio; SGA – small for gestational age

* Hazard ratios from Cox regression

† Adjusted for age at delivery, parity, year of delivery and block group socioeconomic status, homicide, and population size, with interaction of race/ethnicity with exposure, parity, and age at delivery (interaction terms determined from backwards elimination)

‡ Odds ratios from generalized estimating equations; small for gestational age defined as birthweight <10th percentile for gestational age and sex

§ Adjusted for age at delivery, parity, year of delivery and block group socioeconomic status, homicide, and population size, with interaction of race/ethnicity with exposure, parity, age, and block group socioeconomic status (interaction terms determined from backwards elimination)

Table S3. Associations between exposure to police excessive force complaints in the block group (≥1) in the year leading up to delivery and adverse outcomes in the pregnancy cohort, controlling for violent crime rate rather than homicide exposure (n=67,976 deliveries).

	**Hispanic** (n=13,726)		**Black** (n=7,974)		**White** (n=40,483)		**Asian** (n=5,793)	
	HR	95% CI	HR	95% CI	HR	95% CI	HR	95% CI
**Preterm delivery (<37 weeks)** *								
Unadjusted	0.93	0.78, 1.11	1.29	1.13, 1.46	1.08	0.96, 1.21	0.93	0.71, 1.22
Adjusted†	0.92	0.77, 1.10	1.20	1.05, 1.36	1.02	0.91, 1.14	0.95	0.72, 1.25
**SGA infant**‡	OR	95% CI	OR	95% CI	OR	95% CI	OR	95% CI
Unadjusted	0.92	0.76, 1.10	1.14	0.99, 1.31	1.03	0.92, 1.16	1.17	0.95, 1.44
Adjusted†	0.87	0.72, 1.06	1.16	1.00, 1.34	1.02	0.90, 1.15	1.12	0.91, 1.38

Abbreviations: CI – confidence interval; HR – hazard ratio; OR – odds ratio; SGA – small for gestational age

* Hazard ratios from Cox regression

† Adjusted for age at delivery, parity, year of delivery and block group socioeconomic status, violent crime rate, and population size

‡ Odds ratios from generalized estimating equations; small for gestational age defined as birthweight <10th percentile for gestational age and sex

Table S4. Associations between exposure to police excessive force complaints in the block group and preterm delivery in the pregnancy cohort using survival analysis with a time-dependent variable for exposure to police complaints (extended Cox regression), stratified by race/ethnicity (n=67,976 deliveries).

	**Hispanic** (n=13,726)		**Black** (n=7,974)		**White** (n=40,483)		**Asian** (n=5,793)	
	HR	95% CI	HR	95% CI	HR	95% CI	HR	95% CI
**Preterm delivery (<37 weeks)**								
Unadjusted	1.01	0.83, 1.23	1.21	1.05, 1.38	1.08	0.95, 1.23	0.95	0.70, 1.28
Adjusted*	1.01	0.83, 1.22	1.12	0.97, 1.29	1.04	0.92, 1.18	0.93	0.69, 1.26
**Early preterm delivery (<34 weeks)**								
Unadjusted	0.98	0.70, 1.36	1.28	1.05, 1.57	1.00	0.77, 1.31	1.04	0.66, 1.64
Adjusted*	0.99	0.70, 1.38	1.20	0.98, 1.47	0.99	0.76, 1.29	1.02	0.64, 1.62

Abbreviations: CI – confidence interval; HR – hazard ratio

* Adjusted for age at delivery, parity, year of delivery, and block group socioeconomic status, homicide (time-dependent covariate), and population size

Table S5. Associations between exposure to police excessive force complaints in the block group and pregnancy outcomes using block group fixed-effects (stratified Cox regression or conditional logistic regression), stratified by race/ethnicity.

	Hispanic*		Black†		White‡		Asian§	
**Preterm delivery**	HR	95% CI	HR	95% CI	HR	95% CI	HR	95% CI
Unadjusted	0.94	0.77, 1.14	1.23	1.06, 1.43	1.02	0.90, 1.16	1.06	0.77, 1.47
Adjusted‖	0.93	0.77, 1.13	1.18	1.02, 1.38	1.00	0.88, 1.13	0.99	0.71, 1.38
**SGA infant**	OR	95% CI	OR	95% CI	OR	95% CI	OR	95% CI
Unadjusted	0.87	0.70, 1.09	1.14	0.95, 1.37	0.99	0.86, 1.13	1.09	0.83, 1.43
Adjusted‖	0.87	0.69, 1.08	1.15	0.95, 1.40	1.04	0.90, 1.19	1.16	0.88, 1.54

Abbreviations: CI – confidence interval; HR – hazard ratio; OR – odds ratio; SGA – small for gestational age

* Preterm delivery: 9,444 individuals in 660 block groups; SGA infant: 8,840 individuals in 592 block groups

† Preterm delivery: 5,457 individuals in 579 block groups; SGA infant: 5,225 individuals in 549 block groups

‡ Preterm delivery: 36,520 individuals in 720 block groups; SGA infant: 36,211 individuals in 690 block groups

§ Preterm delivery: 3,419 individuals in 217 block groups; SGA infant: 3,977 individuals in 278 block groups

‖ Adjusted for age at delivery, parity, year of delivery, and block group socioeconomic status, homicide, and population size

Table S6. Associations between exposure to police excessive force complaints in the block group in the year leading up to delivery and preterm delivery or small for gestational age (SGA) infant for individuals with multiple deliveries during the study period, stratified by race/ethnicity (n=12,986).

	**Hispanic** n=2,544*		**Black** n=1,103†		**White** n=8,328‡		**Asian** n=1,011§	
**Preterm delivery**‖	HR	95% CI	HR	95% CI	HR	95% CI	HR	95% CI
Unadjusted	0.85	0.56, 1.28	1.28	0.89, 1.84	1.11	0.86, 1.43	1.31	0.64, 2.69
Adjusted	0.82	0.54, 1.25	1.28	0.87, 1.90	1.03	0.79, 1.34	1.13	0.53, 2.42
**SGA infant**‖	OR	95% CI	OR	95% CI	OR	95% CI	OR	95% CI
Unadjusted	1.37	0.83, 2.25	1.69	1.06, 2.67	1.44	1.03, 2.01	1.08	0.60, 1.94
Adjusted	1.09	0.64, 1.84	1.26	0.76, 2.08	1.18	0.81, 1.73	0.71	0.36, 1.40

Abbreviations: CI: confidence interval; HR: hazard ratio; OR: odds ratio; SGA: small for gestational age

* 399 with ≥1 PTD (89 of which vary in exposure); 286 with variation in SGA infant (64 also vary in exposure)

† 252 with ≥1 PTD (113 of which vary in exposure); 195 with variation in SGA infant (78 also vary in exposure)

‡ 958 with ≥1 PTD (237 of which vary in exposure); 755 with variation in SGA infant (145 also vary in exposure)

§ 139 with ≥1 PTD (31 of which vary in exposure); 152 with variation in SGA infant (46 also vary in exposure)

‖ Results from stratified Cox regression (preterm delivery) or conditional logistic regression (SGA infant). Models adjusted for age at delivery, birth order, and block group socioeconomic status, homicide, and population size.

Table S7. Associations between exposure to police excessive force complaints in the census tract (≥1) in the year leading up to delivery and adverse outcomes in the pregnancy cohort, stratified by race/ethnicity (n=67,976 deliveries).

	**Hispanic** (n=13,726)		**Black** (n=7,974)		**White** (n=40,483)		**Asian** (n=5,793)	
	HR	95% CI	HR	95% CI	HR	95% CI	HR	95% CI
**Preterm delivery (<37 weeks)** *								
Unadjusted	0.94	0.82, 1.07	1.30	1.15, 1.48	1.02	0.94, 1.12	0.92	0.77, 1.10
Adjusted†	0.92	0.80, 1.05	1.17	1.03, 1.33	0.99	0.90, 1.09	0.90	0.73, 1.11
**Early preterm delivery (<34 weeks)** *								
Unadjusted	0.84	0.69, 1.02	1.34	1.13, 1.60	0.93	0.81, 1.08	0.90	0.66, 1.24
Adjusted†	0.83	0.67, 1.03	1.22	1.02, 1.46	0.92	0.79, 1.07	0.86	0.61, 1.22
**SGA infant**‡	OR	95% CI	OR	95% CI	OR	95% CI	OR	95% CI
Unadjusted	0.95	0.83, 1.08	1.16	1.03, 1.32	0.98	0.89, 1.09	1.15	0.96, 1.38
Adjusted†	0.94	0.82, 1.08	1.21	1.06, 1.39	0.99	0.90, 1.10	1.05	0.88, 1.25

Abbreviations: CI – confidence interval; HR – hazard ratio; OR – odds ratio; SGA – small for gestational age infant

* Hazard ratios from Cox regression

† Adjusted for age at delivery, parity, year of delivery and census tract socioeconomic status, homicide frequency, neighborhood stability, and population size

‡ Odds ratios from generalized estimating equations; small for gestational age defined as birthweight <10th percentile for gestational age and sex (*41*)

Table S8. Parameter estimates for fully adjusted model of exposure to police excessive force complaints in the block group (top decile) and incident cardiovascular disease among women in the CVD cohort, stratified by race (n=6,773).

	**Black** (n=2,704)		**White** (n=4,069)	
	β	95% CI	β	95% CI
EFP complaint	0.35	0.11, 0.58	0.24	-0.08, 0.57
Smoking status*				
Non-smoker	*Ref*		*Ref*	
Current smoker	0.41	0.17, 0.65	0.49	0.20, 0.77
Former smoker	-0.15	-0.39, 0.10	0.14	-0.06, 0.33
Body mass index*				
<25 kg/m^2	*Ref*		*Ref*	
25-30 kg/m^2	0.27	-0.05, 0.59	0.14	-0.09, 0.36
>30 kg/m^2	0.28	-0.01, 0.56	0.13	-0.10, 0.37
Hypertension*	0.43	0.19, 0.67	0.18	-0.06, 0.41
Diabetes*	0.42	0.20, 0.64	0.68	0.36, 1.00
Homicide	0.07	-0.17, 0.31	0.42	-0.15, 0.99
BG SES	-0.10	-0.23, 0.02	-0.13	-0.27, 0.01
BG population	-0.04	-0.15, 0.07	-0.02	-0.14, 0.10
Year of index visit	-0.08	-0.11, -0.04	-0.05	-0.08, -0.02

Abbreviations: BG SES – block group socioeconomic status; BG population – block group total population (per 1,000); CI – confidence interval; EFP complaint – exposure to complaint about excessive force by police

* All based on index visit

Table S9. Associations between exposure to police excessive force complaints in the block group (top decile) and development of incident cardiovascular disease among women in the CVD cohort, controlling for exposure to violent crime, stratified by race (n=6,773).

	**Black** (n=2,704)		**White** (n=4,069)	
	HR	95% CI	HR	95% CI
Model 1*	1.64	1.30, 2.06	1.29	0.92, 1.80
Model 2†	1.36	1.08, 1.72	1.21	0.91, 1.61
Model 3‡	1.40	1.11, 1.77	1.17	0.90, 1.53

Abbreviations: CI – confidence interval; HR – hazard ratio

* Unadjusted model

† Adjusted for year of index visit and block group socioeconomic status, violent crime rate, and population size

‡ Additionally adjusted for smoking status, body mass index, hypertension, and diabetes, all at the index visit

Table S10. Associations between exposure to police excessive force complaints in the block group (top decile) and development of incident cardiovascular disease among women in the CVD cohort, restricted to those with at least 5 years of follow up, stratified by race (n=4,908).

	**Black** (n=1,999)		**White** (n=2,909)	
	HR	95% CI	HR	95% CI
Model 1*	1.70	1.25, 2.30	1.40	0.86, 2.26
Model 2†	1.51	1.09, 2.07	1.38	0.82, 2.32
Model 3‡	1.44	1.04, 1.99	1.39	0.84, 2.29

Abbreviations: CI – confidence interval; HR – hazard ratio

* Unadjusted model

† Adjusted for year of index visit and block group socioeconomic status, homicide frequency, and population size

‡ Additionally adjusted for smoking status, body mass index, hypertension, and diabetes, all at the index visit

Table S11. Associations between decile of exposure to police excessive force complaints[*] in the block group and development of incident cardiovascular disease among women in the CVD cohort, stratified by race (n=6,773).

	Black (n=2,704)		White (n=4,069)	
	HR	95% CI	HR	95% CI
Model 1[†]				
<70th	*Reference*		*Reference*	
70th – 80th	1.03	0.77, 1.37	0.86	0.59, 1.25
80th – 90th	1.30	0.99, 1.70	1.35	0.94, 1.87
≥90th	1.72	1.36, 2.19	1.30	0.93, 1.83
Model 2[‡]				
<70th	*Reference*		*Reference*	
70th – 80th	0.95	0.70, 1.29	0.80	0.55, 1.18
80th – 90th	1.18	0.89, 1.58	1.31	0.93, 1.84
≥90th	1.51	1.17, 1.95	1.30	0.91, 1.86
Model 3[§]				
<70th	*Reference*		*Reference*	
70th – 80th	0.97	0.72, 1.31	0.81	0.56, 1.18
80th – 90th	1.21	0.91, 1.61	1.25	0.90, 1.75
≥90th	1.47	1.14, 1.90	1.28	0.92, 1.80

Abbreviations: CI – confidence interval; HR – hazard ratio

[*] Cutoffs for deciles of exposure: 70th percentile: 0.22 complaints per year; 80th percentile: 0.32 complaints per year; 90th percentile: 0.56 complaints per year

[†] Unadjusted model

[‡] Adjusted for year of index visit and block group socioeconomic status, homicide frequency, and population size

[§] Additionally adjusted for smoking status, body mass index, hypertension, and diabetes, all at the index visit

Table S12. Associations between exposure to police excessive force complaints in the census tract (top decile, ≥1.57/year) and development of incident cardiovascular disease among women in the CVD cohort, stratified by race (n=6,773).

	**Black** (n=2,704)		**White** (n=4,069)	
	HR	95% CI	HR	95% CI
Model 1*	1.96	1.36, 2.82	1.52	0.85, 2.69
Model 2†	2.04	1.52, 2.75	1.55	0.78, 3.09
Model 3‡	2.02	1.50, 2.72	1.56	0.82, 2.96

Abbreviations: CI – confidence interval; HR – hazard ratio

* Unadjusted model

† Adjusted for year of index visit and census tract socioeconomic status, homicide frequency, and population size

‡ Additionally adjusted for smoking status, body mass index, hypertension, and diabetes, all at the index visit

Table S13. ICD codes for outcomes and covariates in the CVD cohort.

Outcome	ICD9 codes	ICD10 Codes
Ischemic heart disease	410, 411, 412, 413, 414, 429.2	I21.09, I22.0, I21.01, I21.02, I21.19, I22.1, I21.11, I21.29, I22.8, I21.4, I22.2, I21.21, I21.3, I22.9, I24.1, I20.0, I25.110, I25.700, I25.710, I25.720, I25.730, I25.750, I25.760, I25.790, I24.0, I24.8, I24.9, I25.2, I20.8, I20.1, I20.9, I25.111, I25.118, I25.119, I25.701, I25.708, I25.709, I25.711, I25.718, I25.719, I25.721, I25.728, I25.729, I25.731, I25.738, I25.739, I25.751, I25.758, I25.759, I25.761, I25.768, I25.769, I25.791, I25.798, I25.799, I25.10, I25.110, I25.111, I25.118, I25.119, I25.710, I25.711, I25.718, I25.719, I25.810, I25.730, I25.731, I25.738, I25.739, I25.720, I25.721, I25.728, I25.729, I25.790, I25.791, I24.798, I25.799, I25.700, I25.701, I25.708, I25.709, I25.750, I25.751, I25.758, I25.759, I25.811, I25.760, I25.761, I25.768, I25.769, I25.812, I25.3, I25.41, I25.42, I25.82, I25.83, I25.84, I25.5, I25.6, I25.89, I25.9, I23.1, I23.2, I51.0, I23.0, I23.3, I23.6, I23.7, I23.8
Cerebrovascular disease	430, 431, 432, 433, 434, 435, 436, 437, 438, 362.34	G45, G46, H34, I60, I61, I62, I63, I64, I65, I66, I67, I68, I69
Peripheral vascular disorders	093.0, 437.3, 440, 441, 443.1, 443.2, 443.8, 443.9, 447.1, 557.9, 557.1, V43.4	I70, I71, I73.1, I73.8, I73.9, I77.1, I79.0, I79.2, K55.1, K55.8, K55.9, Z95.8, Z95.9
Congestive heart failure	398.91, 402.01, 402.11, 402.91, 404.01, 404.03, 404.11, 404.13, 404.91, 404.93, 425.4, 425.5, 425.6, 425.7, 425.8, 425.9, 428	I09.9, I11.0, I13.0, I13.2, I25.5, I42.0, I42.5, I42.6, I42.7, I42.8, I42.9, I43, I50, P29.0
Diabetes	250.0, 250.1, 250.2, 250.3	E10.0, E10.1, E10.9, E11.0, E11.1, E11.9, E12.0, E12.1, E12.9, E13.0, E13.1, E13.9, E14.0, E14.1, E14.9, E10.2, E10.3, E10.4, E10.5, E10.7, E10.8, E11.2, E11.3, E11.4, E11.5, E11.7, E11.8, E12.2, E12.3, E12.4, E12.5, E12.7, E12.8, E13.2, E13.3, E13.4, E13.5, E13.7, E13.8, E14.2, E14.3, E14.4, E14.5, E14.7, E14.8
Hypertension	401, 402, 403, 404, 405	I10, I11, I12, I13, I15

Equations:

(1) $h(a, X) = h_0(a|a_0)\exp[\sum \beta_i X_i]$

Cox proportional hazards model for pregnancy cohort, where a is gestational age at event or censorship, a_0 is the gestational age at earliest delivery to account for left truncation, and X_i indicates the model covariates (exposure to excessive force by police, age at delivery, parity, year of delivery, homicide exposure, block group socioeconomic status, and block group population size).

(2) $h_g(a, X) = h_{0g}(a|a_0)\exp[\sum \beta_i X_i]$

Stratified Cox model for CVD cohort, where g indicates stratification by 5-year birth cohort, a is age at event or censorship, a_0 is age at index visit to account for left truncation, and X_i indicates model covariates (exposure to excessive force by police, year of index visit, homicide exposure, block group socioeconomic status, block group population size, and smoking status, body mass index, diabetes, and hypertension at the index visit).
